# Consistency analysis of metabolic correlation networks

**DOI:** 10.1186/1752-0509-1-44

**Published:** 2007-09-24

**Authors:** Mark Müller-Linow, Wolfram Weckwerth, Marc-Thorsten Hütt

**Affiliations:** 1 Bioinformatics Group, Department of Biology, Darmstadt University of Technology, 64287 Darmstadt, Germany; 2 Max-Planck-Institute of Molecular Plant Physiology, Science Park Golm, 14424 Potsdam, Germany; 3 School of Engineering and Science, Jacobs University Bremen, 28759 Bremen, Germany

## Abstract

**Background:**

Metabolic correlation networks are derived from the covariance of metabolites in replicates of metabolomics experiments. They constitute an interesting intermediate between topology (i.e. the system's architecture defined by the set of reactions between metabolites) and dynamics (i.e. the metabolic concentrations observed as fluctuations around steady-state values in the metabolic network).

**Results:**

Here we analyze, how such a correlation network changes over time, and compare the relative positions of metabolites in the correlation networks with those in established metabolic networks derived from genome databases. We find that network similarity indeed decreases with an increasing time difference between these networks during a day/night course and, counter intuitively, that proximity of metabolites in the correlation network is no indicator of proximity of the metabolites in the metabolic network.

**Conclusion:**

The organizing principles of correlation networks are distinct from those of metabolic reaction maps. Time courses of correlation networks may in the future prove an important data source for understanding these organizing principles.

## Background

In molecular biology, the consideration of biochemical processes as elements in an abstract network has become more and more important in the last few years [1,2]. These network-oriented approaches bridge the gap between single units and collective behavior. Metabolism is, in a sense, the mediator between organisms and their environment. Resources, external conditions (represented by control parameters like temperature and concentrations of external agents) and energy all affect the organism via its metabolism. At the same time, metabolism is a key field of application of network biology. The classical approach considers metabolic networks as the pattern of connections of metabolites via enzyme-driven reactions. In this way, reaction networks are straightforward abstractions of what is commonly known as metabolic pathway maps. Global structural properties [3-6], statistical parameters like the degree distribution [3,7] and the diameter [3,8], and local properties like the motif content [9] are well studied. In combination with elementary flux mode analysis [10-14], possible routes between different metabolites are quantified within a metabolic map, while flux balance analysis (FBA) [15,16] is suitable to predict the whole-cell behavior by adding constraints to the regulation of metabolic transformations. These theoretical approaches constitute important steps towards dynamics and have the potential to elucidate the fundamental link between topology and dynamics even further. Particularly the recent work by Almaas et al. [17] points in this direction.

Other types of metabolic networks have been established as well in recent time as the orthogonal networks, where enzymes are connected to each other when they share a common metabolite [18], and the correlation networks. In correlation networks a connection between two metabolites (nodes) represents an above-threshold correlation in metabolite concentrations.

Due to their quality of being derived from metabolic concentrations, they constitute an interesting intermediate between topology and dynamics. Here we study the compatibility of this intermediate with its two antipodes: the topological structure given by the network of metabolic reactions and the dynamic behavior given by the time evolution of the correlations between metabolites.

**Figure 1:**
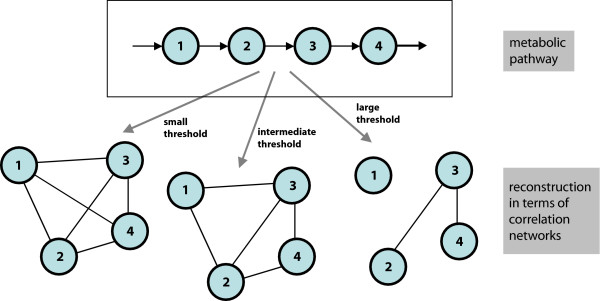
**Basic scheme of relations between the two networks**. Schematic example of the relation between metabolic reaction and metabolic correlation networks. We assume a hypothetical scenario, where a simple chain of biochemical reactions produces strong correlations between the metabolites, which however decrease with the distance of two metabolites in the chain. The bottom part of the figure gives the (hypothetical) reconstructed correlation networks for different regimes of the threshold parameter *κ*.

The different relations between metabolites in both types of networks are illustrated in Fig. 1, which gives a qualitative view in an idealized situation, where all correlations between metabolites are produced by the reactions in a linear four-element chain. In this schematic example, the correlations are assumed to be high for immediate neighbors in the chain and slightly lower at higher distances. One sees that for small and intermediate thresholds in the correlation matrix the reconstructed network tends to compact the linear chain, while higher threshold values may break the chain. The precise pattern, how correlations decay along the chain, depends on details of the enzyme kinetics [19]. Though this concept offers an intuitive explanation for a distribution of correlation coefficients it is reasonable to ask how these correlations are influenced in a more complex network structure taking other aspects into account like superior regulatory mechanisms.

Such correlation networks have been reconstructed both from experimental data [20,21] and from numerical simulations [19]. Steuer et al. [19] used a stochastic system of linear equations based on an underlying metabolic network of biochemical reactions to discover correlations between metabolite fluctuations around a steady-state. This system is related to the metabolic control analysis (MCA) [10,22-26], which also served as a groundwork for linear and non-linear perturbation studies of Camacho et al. [27] and Vance et al. [28]. Camacho et al. [27] point out that different sources of variability can, in principle, lead to the observed correlations. All these studies show that the relation between metabolic correlation networks and the actual network given by the metabolic reactions is far from trivial. We therefore look at the similarity of these two graphs with simple topological tools asking, if two nodes are close to each other in the one network, when they are close in the other.

From the experimental point of view, metabolomic technologies provide widely-used tools to identify compounds in biological samples and to describe the current state of a system [29,30]. For details on the quantitative and qualitative analysis of the single metabolites see Weckwerth and Morgenthal [31] and Kell [32]. More than 1000 different compounds have been isolated and identified in a single tissue in this way.

The studies on different samples revealed strong correlations between certain pairs of metabolites, while most other combinations displayed little or no correlation. Such a correlation profile may serve as a basis for the construction of a metabolic correlation network. Because anti-correlations might have a physiological cause as well, they are treated equally to positive correlations. Beyond the identification of compounds via the concept of correlation networks, metabolomics is also capable to describe physiological processes in consequence of development and changing environmental conditions, since tissue samples can be analyzed at any point of time.

The interrelation between the architecture of a metabolic pathway map and the dynamic processes taking place upon it has sparsely been studied. Results concern the distribution of node degrees and of metabolic fluxes which have both been found to be scale-free [2,3,33] and the metabolic fluxes within the core of the metabolic network of *E. coli*, *H. pylori*, and *S. cerevisiae *[17], but most other studies deal with artificial networks. In the case of metabolic networks one observes discrepancies between theoretical graphs and the real-world networks' topologies. One key publication on gene expression data in yeast [34] indicates that metabolic reaction networks display both scale-free and exponential degree distributions depending on whether one takes all potential paths or particular realizations under certain conditions into account. Furthermore, the biochemical modules derived from experimental data deviate from proposed modules in theoretical reaction networks. Here we will not focus on topological properties of metabolic correlation networks, but look at them as a mediator between topology and dynamics. Since a correlation network represents a dynamic aspect of the metabolic network, it is suitable for comparison with its topological counterpart.

## Results and discussion

### Time consistency of metabolic correlation topologies

When analyzing the metabolite correlations in different plant samples at different sampling points in time one has to consider three potential contributions to the data: Short-term fluctuations in the metabolites' concentrations represented by all plant samples at a given time point may either reflect intrinsic noise or may originate from plant-to-plant variability. Lastly, systematic changes of the steady-state correlation networks over time along a diurnal cycle may contribute to the data. Here we want to find out, whether the (steady-state) correlation networks obtained from different time points are systematically similar. Two main effects can be expected: (1) network similarity should on average be higher for networks at neighboring time points compared to more distant time points, (2) day-night and night-day transitions should be associated with substantially lower network similarity (compared to neighboring time points at constant illumination).

**Figure 2:**
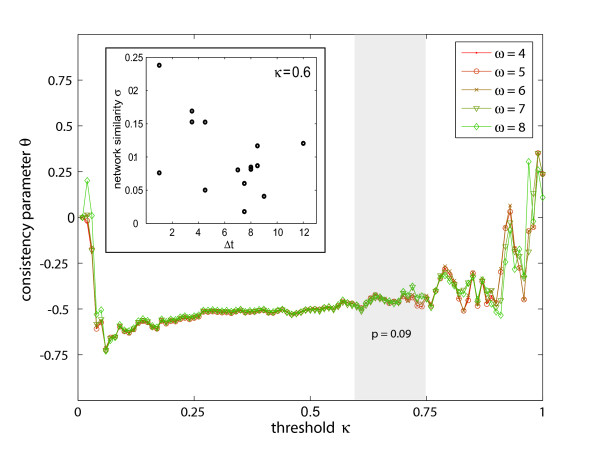
**Temporal consistency within the metabolic correlation networks**. The temporal systematics of different correlation networks has been determined via the consistency parameter *θ *as a function of parameter *κ*. Different values of parameter *ω *(see Methods) have been investigated. Negative values of *θ *indicate a strong anti-correlation between the similarity of two networks and the proximity of the sampling points in time, i.e. similar correlation networks are temporally close to each other. The regime enclosed by the gray-shaded box describes the statistically reliable region of *κ *with an average *p*-value of 0.09. The inset displays the correlation between the time difference Δ*t *and network similarity *σ *for a selected value of threshold *κ*.

In the first step of our analysis, we investigated this temporal property by studying the similarity *σ *of different correlation networks (defined in the Methods section) as a function of their relative positions in time. We want to see, if the network similarity depends on the time difference Δ*t *between the corresponding sampling points. Due to the non-uniform sampling in time and the strong dependence of *σ *on the threshold *κ*, it is convenient to restrict the analysis to the question, whether a conclusion exists between the network similarity *σ *and the time difference Δ*t*. In the following, the correlation coefficient between network similarity *σ *and time difference Δ*t *is denoted by *θ *= *θ*(*κ*), which is a measure of the temporal systematics of the correlation networks. For the evaluation of Δ*t*, we assumed a periodic rhythm of 24 hours, i.e. the intervals of all sampling points with more than 12 hours difference were recalculated according to a 24 h cycle. The interval between 4 h light and 15.5 h dark, for example, is 19.5 h (linear) and 4.5 h (circular). For each sampling point in time we varied the threshold *κ *in steps of 0.01. Note that when *κ *is varied between 0 and 1, connectivity varies accordingly. For each value of *κ*, the similarity of each pair of correlation networks (resulting from the 6 sampling points) was determined (see Methods for details). The correlation coefficient *θ *is then determined by forming pairs of *σ *and Δ*t*. Fig. 2 depicts the results for different values of the data reliability *ω *(see Methods). The consistency of all curves suggests a high robustness of the results to the choice of data. Over a wide range of *κ*, *θ *exhibits strong anti-correlated values, which indicates a higher network similarity the closer the sampling points lie in time. For values of *κ *< 0.1 all networks are approximately the same, thus losing all correlations, while for higher values of *κ *the emerging fluctuations of *θ *can be regarded as a consequence of a low connectivity and network fragmentation and the corresponding loss of correlation data. Significance issues enter Fig. 2 on two levels. First, the small number of replicates, which forms the basis of the covariance matrix, provides a lower significance limit of the threshold *κ*. We used a *p*-value of 0.05 for this, leading to *κ *> 0.6. Second, above a certain level in *κ *the network is fragmented and some pair-wise distances are no longer defined. For our data fragmentation sets in for *κ *≈ 0.75. In Fig. 2 and the consecutive figures we highlight the range of *κ *obeying both criteria. Analyzing statistically significant deviations from zero of *θ *in Fig. 2 for this range of *κ*, we find a *p*-value *p *< 0.09. The scatter diagram (inset in Fig. 2) exemplarily displays the relation between the time difference Δ*t *and network similarity *σ*. For *κ *= 0.6 the corresponding correlation coefficient (i.e. the consistency parameter *θ*) is *θ *= -0.48. If one repeats this consistency analysis with correlation networks taken at identical connectivities (as opposed to identical thresholds *κ*, as in Fig. 2), we also observe a systematic deviation of *θ *from zero in the interesting range of *κ*. In order to rule out the influence of any intrinsic network properties on *σ *we also checked that the consistency parameter *θ *is close to zero for the relevant range of *κ *when we maximally randomize the correlation networks.

Surprisingly, we find a high network similarity for the night-day transition and a low similarity for the day-night transition. Understanding this would, however, require more data. Omitting day-night transitions from this analysis and determining the consistency parameter *θ *at 0.6 <*κ *< 0.75 consequently yields a more pronounced result with comparable *p*-values.

### Are correlation networks and metabolic reaction networks related?

On the basis of the data set of Ma and Zeng [35] and the data set of Weckwerth et al. [20,21], 39 common compounds have been identified, which are present in both networks. This value primarily depends on the number of identified compounds in the metabolomic analysis. Unidentified compounds are systematic signals in the metabolomic data, which cannot be unambiguously associated with a node in the reaction network. The effect of unidentified metabolites is difficult to estimate. Whether they influence the length scale (an average distance of identified metabolites) or not mainly depends on the individual structure of the network. The known metabolites show approximately the same distribution of pair distances as the complete reaction network and can therefore be regarded as a representative subset of the whole graph in this property. We also checked the influence of unknown nodes on the network similarity *σ *for two Erdős-Rényi (ER) random graphs [36] (with a starting value of *σ *= 0.3) when using less and less nodes for this computation. Similarity *σ *displays only a marginal dependency on the number of contributing nodes even if half of the nodes are used.

The common metabolites may serve as a basis for the computation of the pair distances, the statistical parameter already used in the previous section to determine the relation between two networks. This compound list primarily consists of two biochemical substance classes, namely the carbohydrates and amino acids and their corresponding derivatives (for details on the available amino acids and carbohydrates: see Table 1). There are a few compounds, which are assigned to the citric acid cycle, the photosynthesis, and the phospholipid and glycolipid metabolism, respectively.

**Table 1:** Selected common compounds from the metabolic reaction and the metabolic correlation networks

Amino acids		Carbohydrates	
1	*beta*-Alanine	15	D-Ribose
2	L-Asparagine	16	D-Fructose
3	L-Aspartate	17	D-Mannose
4	L-Glutamate	18	D-Glucose
5	L-Glutamine	19	D-Fructose-6-phosphate
6	Glycine	20	D-Glucose-6-phosphate
7	L-Isoleucine	21	Sucrose
8	L-Methionine	22	Maltose
9	L-Serine	23	Raffinose
10	L-Threonine	24	D-Glucose-1-phosphate
11	L-Tyrosine		
12	L-Valine		
13	L-Phenylalanine		
14	L-Cysteine		

List of compounds belonging to the class of amino acids and carbohydrates which have been found in both metabolic networks of *A. thaliana*.

**Figure 3:**
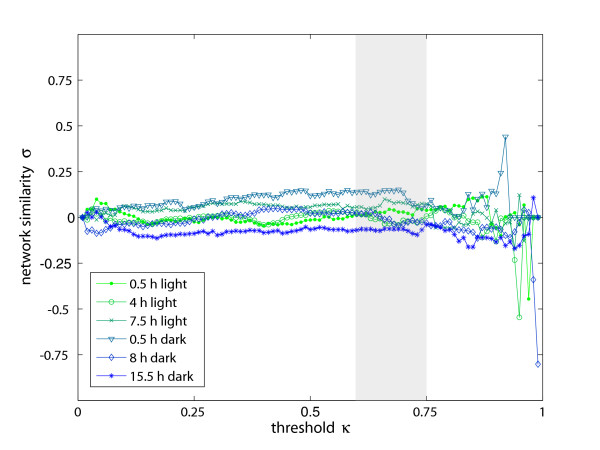
**Relation of metabolic reaction and metabolic correlation networks (pair distances)**. Comparison of metabolic reaction networks and metabolic correlation networks via the pairwise distances between nodes. The similarity between both types of networks (correlation network at *ω *= 8) has been determined for different sampling points and 0 <*κ *< 1. Values around zero indicate, that there is no correlation between these types of metabolic networks. The regime enclosed by the gray-shaded box describes the statistically reliable region of *κ*.

**Figure 4:**
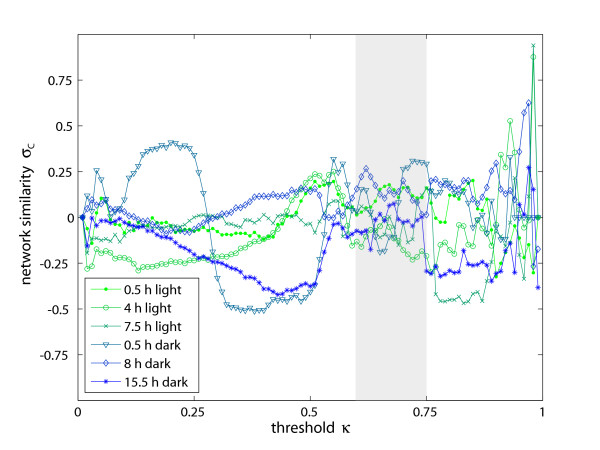
**Relation of metabolic reaction and metabolic correlation networks (centrality)**. Another method of quantifying network similarity uses an individual node parameter, the centrality for comparison of both types of metabolic networks. Computing the similarity of the two networks based upon this parameter, which is the average path length of all shortest paths connecting a node to all other nodes, shows no significant relation between metabolic reaction and correlation networks. The regime enclosed by the gray-shaded box describes the statistically reliable region of *κ*.

Again, we generated correlation networks of varied connectivity for each sampling point in time by adjustment of *κ *between 0 and 1. In the following, only the results for *ω *= 8 are discussed, since only marginal differences were observed for *ω *< 8. Results of the network similarity *σ *for all sampling points are depicted in Fig. 3. Limits for the significance analysis were applied as described before. In this range of *κ *two sampling points display minor positive correlations and one sampling point minor negative correlations (with average *p*-values of 0.04, 0.07, and 0.08). The other three sampling points display no systematic deviation from zero (with *p*-values above 0.45). Thus, taking all sampling points together the results suggest no correlation between the two network types. Due to the three sampling points failing our significance analysis, however, we analyze this correlation also by means of another network property, namely the centrality *σ*_*C *_[4,35] of all common nodes. This topological parameter was tested accordingly (Fig. 4) and, here, no significant deviations from zero similarity (with average *p*-values above 0.3) and therefore from the pair distance results were observed.

### Analysis of module-module interaction

**Figure 5:**
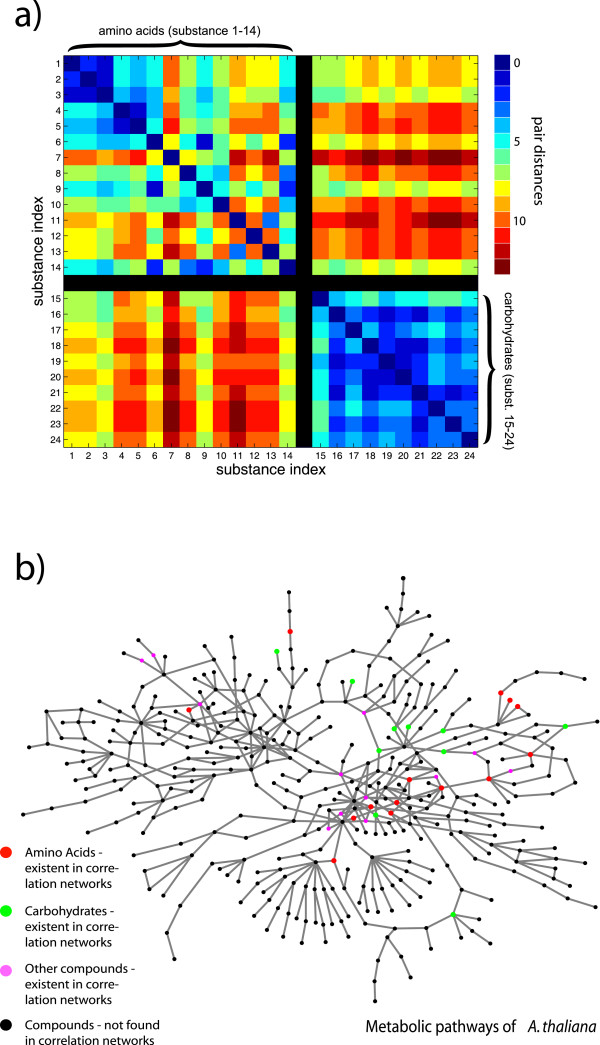
**Selected substances in the metabolic reaction network of A. thaliana**. We investigated the common compounds of both types of metabolic networks belonging to the classes of amino acids and carbohydrates, respectively. a) Color-coded are the pair distances of these metabolites. As before, the pair distance is the number of connections in the shortest path between two compounds. Most of the carbohydrates are characterized by small internal pair distances, while there are more links needed to connect them to the group of amino acids, which display a relatively small average internal pair distance as well. b) This graph representation shows the giant component of the metabolic reaction network of *A. thaliana*. All metabolites, which have also been found in the correlation network, are color-coded according to their biochemical class (see Fig. 5a).

**Figure 6:**
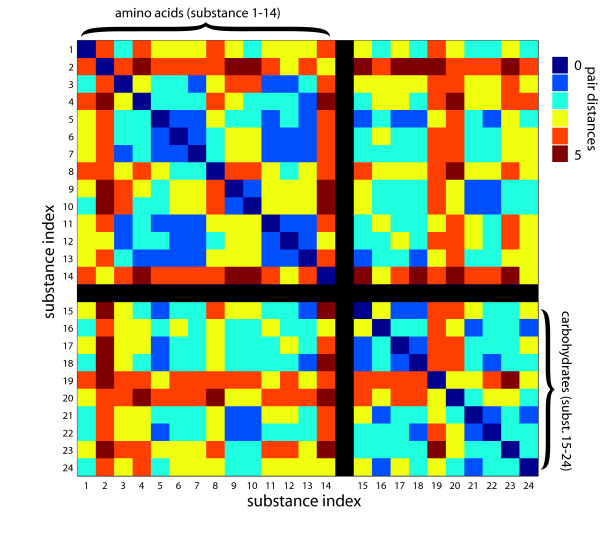
**Amino acids and carbohydrates in a metaboliccorrelation network**. The correlation network of *A. thaliana *displays other distance relations of amino acids and carbohydrates compared to the reaction network in Fig. 5a. This representative example from sampling point 4 h light shows the color-coded pair distances of all metabolites belonging to the biochemical classes of the amino acids and of the carbohydrates. The typical separation of compounds according to their biochemical classes as found in the metabolic reaction network (see Fig. 5a) is not seen in the correlation networks independently of the sampling point and the parameter *ω*(data not shown). A distinct intra-class correlation which has been observed in most of the sampling points exists for Isoleucine(7), Tyrosine(11), and Valine(12). Some sampling points, however, exhibit correlations which hint to inter-class specific relations (e.g. serine(9) and sucrose(21)).

The different distribution of metabolites in both types of networks is best illustrated through the comparison of class-specific compounds. We identified all metabolites in the networks, which belong to the group of amino acids and carbohydrates (Table 1), respectively, and calculated all pair distances within these groups. We checked all sampling points, different values of *ω*, and adjusted *κ *in a way to obtain sparsely connected networks, which in their connectivity resemble their topological counterpart. The metabolic reaction network (Fig. 5a) exhibits a distinct clustering within the biochemical classes, especially for the carbohydrates (with an average pair distance of 3.1). Fig. 5b depicts the distribution of these selected compounds across the reaction network. The correlation networks (Fig. 6) display no significant difference between the intra-class and inter-class specific pair distances, if one takes all compounds into account. We performed a UPGMA cluster analysis on the distance matrices of the correlation networks in order to find the inter-class and intra-class specific clusters of metabolites with special regard to their day and night time-specific emergence. To insure that more than 95 % of the selected metabolites lie within the giant component of the network we restricted this analysis to values of 0.75 <*κ *< 0.8. We recovered a prescribed number *n*_*m *_= 5 of clusters each with at least two metabolites by horizontally cutting the tree at a certain hight. Therefore, one has to analyze the dependence of the cluster predictions on threshold variation. That way, several omnipresent groups of metabolites could be identified (e.g. a cluster of the amino acids 7, 11, and 12). Others appeared either at day or at night. Within the night samples, there were two distinct groups one solely containing metabolites of the class of carbohydrates (19, 20, and 24) and one inter-class specific group (4, 16, and 23), while the day samples contained several intra-class specific clusters (e.g. 21 and 22) and one inter-class specific cluster (9 and 21). All metabolites in the inter-class specific clusters display a pair distance of at least 7 in the reaction network.

## Conclusion

In this work we investigated systematically the relationship between metabolic correlation networks and genome-wide predicted reaction networks. In recent studies we investigated how correlation networks are causally connected to the underlying biochemical reaction network and its regulation [19,20,30,37]. However, all these studies revealed the discrepancy between predicted pathway connectivity and a simple extrapolation of these hypothetical networks to correlation networks. We detected overall changes in the correlation network topology, which are rather based on specific enzyme activity alterations [21,30]. Therefore a systematic comparison of predicted pathway map structure and the experimental metabolite covariance is crucial. Based on the prediction that alterations in biochemical regulation will change the correlation network structure [20] we have investigated different correlation network structures during a diurnal time course in Arabidopsis plant leaf samples. Intriguingly, the structure of these networks did not match the predicted pathway connectivity in plant metabolism, but nevertheless varies systematically in time. Moreover, compared to theoretical pathways in Arabidopsis the correlation network structure revealed different and novel clusters of compounds such as the correlation of aromatic amino acids and housekeeping sugars which is not predicted by the topology of theoretical pathways (Fig. 6). This fact supports our hypothesis that the correlation network largely reflects biochemical regulation. Particularly this observation that the systematics of correlation networks are somewhat orthogonal to those contained in pathways networks, suggests that the systematic and differential analysis of metabolite correlation network structures and metabolite covariances will in the future lead to novel insights into biochemical regulation and regulatory hubs in the *in vivo *system.

## Methods

### Construction of correlation networks

The analysis of the correlation data and the respective correlation networks was based on the experimental data sets of Weckwerth et al. [20,21]. In their studies, they recorded the concentrations of 257 different metabolites of *Arabidopsis thaliana*. Ten samples of this plant had been cultivated for 26 days under the same conditions with a diurnal rhythm of 8 h light and 16 h darkness. Data from 6 sampling points (three in each phase of illumination; in detail: 0.5 h, 4 h, and 7.5 h light; 0.5 h, 8 h, 15.5 h dark) were drawn from each plant, resulting in 60 measurements.

In principle, there are two practicable approaches to construct a correlation network from the data given here. Considering long-term fluctuations one would use the plant-specific changes in time given by six concentration values. We preferred a statistically more reliable method which considers the short-term fluctuations provided by 10 different plant samples. Using 6 samples in time instead of 10 plant samples largely limits the statistically reliable range of *κ*, setting the lower boundary of *κ *to a value of 0.82 (for a *p*-value of 0.05). This threshold matches the upper threshold approximately, where all networks tend to fragment.

**Figure 7:**
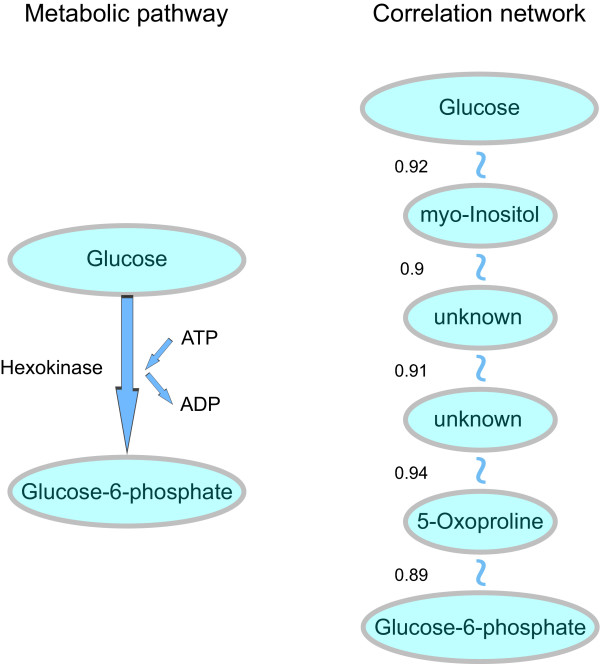
**Example of a shortest path in a metabolic reaction network and a metabolic correlation network**. The transformation of glucose to glucose-6-phosphate in glycolysis is represented by one connection in the pathway network. In this example of a sparsely connected but non-fragmented correlation network (parameter constellation: sampling point 0.5 h light, *ω *= 8 and *κ *= 0.85) the shortest path between this pair of metabolites consists of 5 links (numbers next to the links denote the individual correlation strength of the involved metabolite pairs; here, the correlation strength between glucose and glucose-6-phosphate is 0.13.). The deviation of path lengths shown in this example just demonstrates the possible structural differences within these networks.

Correlation networks were reconstructed from the measured metabolite concentrations by pair-wise combination of the metabolite concentrations. In the correlation network, there is an edge between two compounds, if the absolute value of the correlation coefficient resulting from two concentration vectors exceeds a certain threshold *κ*, i.e. positive correlations and anti-correlations are treated likewise. Fig. 7 displays the connections between glucose and glucose-6-phosphate (G6P) in one of the correlation networks of *Arabidopsis thaliana *[20,21] (Fig. 7 right). In the reaction network both metabolites characterize the first reaction step in glycolysis, which is catalyzed by hexokinase under consumption of ATP (Fig. 7 left). The correlation coefficient of glucose and G6P is close to zero, which means that there is nearly no correlation between these two compounds under the studied conditions. Considering very strong correlations only, multiple links are required to get from the one compound to the other. In the example given in the right-hand part of Fig. 7 the corresponding part of the correlation network is shown for the sampling point 0.5 h with *ω *= 8 and *κ *= 0.85; in this case, the correlation coefficient of glucose and G6P is 0.13. Two alternative shortest paths using 5 links exist here, one is depicted here with the corresponding correlation strengths of each metabolite pair. Some of the compounds have been observed in the experiment but not identified yet.

By construction, the threshold (0 ≤ *κ *≤ 1) regulates the connectivity of a correlation network. For *κ *= 0 one obtains a complete network, i.e. every node is connected to every other node. Occasionally, in the measurements some metabolites have not been registered. In these cases gaps occur in the data matrix. We account for these missing measurements by introducing a second threshold *ω *into our analysis and restrict the range of metabolites in the network to those pairs with at least *ω *measurement results. In this way we qualitatively vary the reliability of the data entering our analysis. We changed this parameter in an interval of 4 ≤ *ω *≤ 8 in order to validate the robustness of the results with regard to the choice of data.

### Data sets of metabolic networks

Various databases based on genomic analysis provide information on metabolic reactions. Among these the KEGG database [38] has been used by several research groups for the reconstruction of metabolic networks. This comparatively reliable group within the biochemical networks opens up a reaction space, which is used for metabolic transformations. Basically two approaches to interpret a reconstructed metabolic network exist so far, which differ in the consideration of ubiquitous compounds like H_2_O, ATP, and NADH, often referred to as current (or currency) metabolites. These hub forming compounds drastically reduce the average path length and, consequently, the pathways in such a metabolic network do not resemble the conventional order of reactions. Ma and Zeng [35] addressed this issue by eliminating all connections from a current metabolite, if the connections represent particular reactions, like the transfer of electrons or of certain functional groups. Additionally, they corrected mistakes and inconsistences in the original data set and checked the reversibility and direction information of each reaction.

The reconstruction of the metabolic network of *A. thaliana*, used in this paper, is based on the data from Ma and Zeng (http://genome.gbf.de/bioinformatics/index.html[35]). This connection table displays 2070 different enzyme-driven transformations summed up over 107 organisms and broken down into reversible and irreversible reactions. A reaction in this table represents a potential connection within the metabolic network, while the existence of a link in a certain organism is indicated by a value greater than or equal to unity for each organism-reaction combination. (This value refers to the number of genes encoding this enzyme.) After cross-linking all metabolites listed in this table, an undirected subnetwork was extracted from this data by conversion of all directed links (irreversible reactions) into undirected links (reversible reactions) and by identification of the giant cluster, which is the largest connected component in a fractionated network. This step from directed to undirected networks for the sake of this comparison is necessary, as the correlation networks are undirected by construction. Thus, the 760 connections (438 reversible and 332 irreversible reactions) in the original data set were reduced to 492 undirected edges within a metabolic network of 404 nodes. This resulting network will be termed metabolic reaction network.

### Similarity analysis of networks

Various methods for the determination of the similarity of two networks exist so far. A prominent example is the graph alignment [39,40], which is a suitable method for related networks. The great variability of the considered networks here asks for other statistical parameters. Thus, the determination of the similarity *σ *will be accomplished via comparison of all pair distances in the networks. The pair distance *δ*_*ij *_is defined as the shortest path between the two nodes *i *and *j*, i.e. the smallest number of links which connects two nodes. Undirected connected networks of size *n *hold a maximum number *m*_*max *_of pair distances of *m*_*max *_= *n*(*n *- 1)/2. This number drops in fractionated networks where the path lengths between nodes in different clusters cannot be calculated. For network comparison, only those pair distances are taken into account, which can be computed in both networks. The similarity *σ *= *σ *(*G*_1_*, G*_2_) between the two networks *G*_1 _and *G*_2 _is the Pearson correlation coefficient of the respective pair distance vectors.

We tested this method both with real networks of different size but similar structure and with artificial graphs of the same size and connectivity which were altered systematically. Therefore, we compared the reaction network of *A. thaliana *with six reaction networks of other eukaryotes from the Ma and Zeng database. Despite their different size (332 <*n *< 625) the networks displayed an average network similarity *σ *of 0.81.

**Figure 8:**
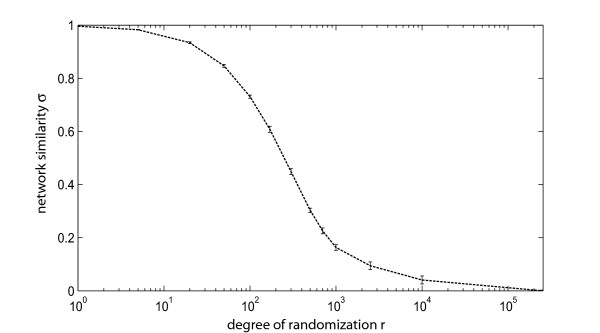
**Example of the similarity analysis in an artificial network**. The network similarity *σ *for an ER graph (*n *= 250 nodes, *m *= 1245 edges) and its rewired versions has been computed as a function of the randomization depth *r *(number of rewired edges). In each randomization step one endpoint of an edge is rewired to another randomly chosen node.

In order to see whether our similarity indicator *σ *is capable of capturing the monotonous decrease in similarity under increasing randomization of one of the networks we analyzed *σ *for an ER graph [36] with *n *= 250 nodes and *m *= 1245 edges and its rewired counterparts as a function of the randomization depth *r *which corresponds to the number of rewired edges (Fig. 8). In each step of this randomization procedure one end of a randomly chosen edge is rewired to new randomly chosen node. With increasing *r *the continuous destruction of the original network structure is reflected in an explicit reduction of the network similarity *σ *until all networks display zero similarity. In our studies on the relation of correlation networks and metabolic reaction networks, we additionally used the centrality *σ*_*C *_[4,35] to compare both types of networks. The centrality *σ*_*C *_of a node is defined as the average path length of all shortest paths connecting this node to all other nodes.

## Competing interests

The author declares that there are no competing interests.

## References

[B1] Alon U (2007). An introduction to systems biology.

[B2] Barabási AL, Oltvai ZN (2004). Network Biology: Understanding the Cell's Functional Organization. Nature Reviews Genetics.

[B3] Jeong H, Tombor B, Albert R, Oltvai ZN, Barabási AL (2000). The large-scale organization of metabolic networks. Nature.

[B4] Wagner A, Fell DA (2001). The small-world inside large metabolic networks. Proc R Soc Lond B.

[B5] Ravasz E, Somera AL, Mongru DA, Oltvai ZN, Barabási AL (2002). Hierarchical organization of modularity in metabolic networks. Science.

[B6] Guimerà R, Amaral LN (2005). Functional cartography of complex metabolic networks. Nature.

[B7] Tanaka R (2005). Scale-Rich Matabolic Networks. Phys Rev Lett.

[B8] Arita M (2004). The metabolic world of *Escherichia coli *is not small. PNAS.

[B9] Milo R, Itzkovitz S, Kashtan N, Levitt R, Shen-Orr S, Ayzenshtat I, Sheffer M, Alon U (2004). Superfamilies of Evolved and Designed Neworks. Science.

[B10] Heinrich R, Schuster S (1996). The regulation of cellular systems.

[B11] Schilling CH, Schuster S, Palsson BO, Heinrich R (1999). Metabolic pathway analysis: basic concepts and scientific applications in the post-genomic era. Biotechnol Prog.

[B12] Schuster S, Dandekar T, Fell DA (1999). Detection of elementary flux modes in biochemical networks: a promising tool for pathway analysis and metabolic engineering. Trends Biotechnol.

[B13] Schuster S, Fell DA, Dandekar T (2000). A general definition of metabolic pathways useful for systematic organization and analysis of complex metabolic networks. Nat Biotechnol.

[B14] Schuster S, Hilgetag C, Woods JH, Fell DA (2002). Reaction routes in biochemical reaction systems: algebraic properties, validated calculation procedure and example from nucleotide metabolism. Math Biol.

[B15] Varma A, Palsson BO (1994). Metabolic flux balancing: basic concepts, scientific and practical use. Biotechnology.

[B16] Edwards JS, Palsson BO (2000). Metabolic flux balance analysis and the in silicio analysis of *Escherichia coli *K-12 gene deletions. BMC Bioinformatics.

[B17] Almaas E, Oltvai ZN, Barabási AL (2005). The Activity Reaction Core and Plasticity of Metabolic Networks. PLoS Comp Biology.

[B18] Horne AB, Hodgman TC, Spence HD, Dalby AR (2004). Constructing an enzyme-centric view of metabolism. Bioinformatics.

[B19] Steuer R, Kurths J, Fiehn O, Weckwerth W (2003). Observing and interpreting correlations in metabolomic networks. Bioinformatics.

[B20] Weckwerth W (2003). Metabolomics in systems biology. Annu Rev Plant Biol.

[B21] Morgenthal K, Wienkoop S, Scholz M, Selbig J, Weckwerth W (2005). Correlative GC-TOF-MS based metabolite profiling and LC-MS based protein profiling reveal time-related systemic regulation of metabolite-protein networks and improve pattern recognition for multiple biomarker selection. Metabolomics.

[B22] Kacser H, Burns JA (1973). The control of flux. Symp Soc Exp Biol.

[B23] Heinrich R, Rapoport SM, Rapoport TA (1977). Metabolic regulation and mathematical models. Prog Biophys Mol Biol.

[B24] Fell DA, Sauro HM (1985). Metabolic control and its analysis. Additional relationships between elasticities and control coefficients. Eur J Biochem.

[B25] Reder C (1988). Metabolic control theory: a structural approach. Theor Biol.

[B26] Heinrich R, Reder C (1991). Metabolic control analysis of relaxation processes. Theor Biol.

[B27] Camacho D, de la Fuente A, Mendes P (2005). The origin of correlations in metabolomics data. Metabol.

[B28] Vance W, Arkin A, Ross J (2002). Determination of causal connectivities of species in reaction networks. PNAS.

[B29] Roessner U, Luedemann A, Brust D (2001). Metabolic profiling allows comprehensive phenotyping of genetically or environmentally modified plant systems. Plant Cell.

[B30] Weckwerth W, Loureiro ME, Wenzel K, Fiehn O (2004). Differential metabolic networks unravel the effects of silent plant phenotypes. PNAS.

[B31] Weckwerth W, Morgenthal K (2005). Metabolomics: from pattern recognition to biological interpretation. DDT.

[B32] Kell DB (2004). Metabolomics and systems biology: making sense of the soup. Curr Opin Microbiol.

[B33] Almaas E, Kovács B, Vicsek T, Oltvai ZN, Barabási AL (2004). Global organization of metabolic fluxes in the bacterium *Escherichia coli*. Nature.

[B34] Ihmels J, Levy R, Barkai N (2004). Principles of transcriptional control in the metabolic network of *Saccharomyces cerevisiae*. Nat Biotechnol.

[B35] Ma H, Zeng AP (2003). Reconstruction of metabolic networks from genome data and analysis of their global structure for various organisms. Bioinformatics.

[B36] Erdős P, Rényi A (1959). On the evolution of random graphs. Publicationes Mathematicae.

[B37] Morgenthal K, Weckwerth W, Steuer R (2006). Metabolomic networks in plants: Transitions from pattern recognition to biological interpretation. Biosystems.

[B38] Ogata H, Goto S, Sato K, Fujibuchi W, Bono H, Kanehisa M (1999). KEGG: Kyoto Encyclopedia of Genes and Genomes. Nucleic Acids Res.

[B39] Berg J, Lässig M (2004). Local graph alignment and motif search in biological networks. PNAS.

[B40] Berg J, Lässig M (2006). Cross-species analysis of biological networks by Bayesian alignment. PNAS.

